# Clinical, Histological, and Genetic Characterization of a Large Cohort of 49 Patients With Nebulin‐Related Congenital Myopathy

**DOI:** 10.1155/humu/4385685

**Published:** 2026-08-17

**Authors:** Yvan de Feraudy, Anthony Maino, Morgane Boedec, Xavière Lornage, Norma B. Romero, Soledad Monges, Fabiana Lubieniecki, Marίa Eugenia Foncuberta, Celine Tard, Claude-Alain Maurage, Marie Csanyi, Pascale Marcorelles, Tanya Stojkovic, Leonard Feasson, Mélanie Fradin, Gaelle Gousse, Vincent Laugel, Marion Masingue, Aleksandra Nadaj-Pakleza, Jorge Alfredo Bevilacqua, NEB study group, John Rendu, Johann Bohm, Valérie Biancalana, Jocelyn Laporte

**Affiliations:** ^1^ Département de Médecine Translationelle, Institut de Génétique et de Biologie Moléculaire et Cellulaire (IGBMC), Cnrs UMR7104, Inserm U1258, Université de Strasbourg, Illkirch-Graffenstaden, France, unistra.fr; ^2^ Pediatric Neurology Department, Reference Centre for Neuromuscular Disorders, Hautepierre Hospital, Strasbourg, France; ^3^ Inserm, U1216, CHU Grenoble Alpes, Grenoble Institut Neurosciences, Univ. Grenoble Alpes, Grenoble, France, univ-grenoble-alpes.fr; ^4^ Myology Institute, Neuromuscular Morphology Unit, Sorbonne Université, INSERM, GHU Pitié-Salpêtrière, Paris, France, inserm.fr; ^5^ Neurology Department, Garrahan Pediatric Hospital, Buenos Aires, Argentina; ^6^ Department of Pathology, Garrahan Pediatric Hospital, Buenos Aires, Argentina; ^7^ Molecular Biology Laboratory, Garrahan Pediatric Hospital, Buenos Aires, Argentina; ^8^ Neurology and Movement Disorders Department, U1172, University Hospital Centre (CHU) de Lille, Reference Centre for Neuromuscular Diseases Nord Est Ile de France, Lille, France; ^9^ North-East Ile-de-France Neuromuscular Pathology Reference Center, University Hospital Center of Lille, Lille, France; ^10^ Department of Pathology, University Hospital Morvan, Brest, France; ^11^ Nord/Est/Ile-de-France Neuromuscular Reference Centre, Pitié-Salpêtrière Hospital, AP-HP, Sorbonne University, Paris, France, sorbonne-universites.fr; ^12^ Myology Unit, Reference Center of Rare Neuromuscular Diseases, Euro-NmD, University Hospital of Saint-Etienne, Saint-Etienne, France, chu-st-etienne.fr; ^13^ Service de Génétique Clinique, Centre de Référence Maladies Rares Anomalies du Développement, CHU de Rennes, Rennes, France, chu-rennes.fr; ^14^ Reference Center of Rare Neuromuscular Diseases, Euro-NmD, University Hospital of Saint-Etienne, Saint-Etienne, France, chu-st-etienne.fr; ^15^ Reference Center for Neuromuscular Diseases NEIdF and ERN EURO-NMD, Neurology Department, University Hospital of Strasbourg, Strasbourg, France, chru-strasbourg.fr; ^16^ Neuromuscular Unit, Department of Neurology and Neurosurgery, Hospital Clínico Universidad de Chile and Clínica Davila Recoleta, Santiago, Chile. Department of Anatomy and Legal Medicina, Faculty of Medicine, Universidad de Chile, Santiago, Chile, uchile.cl; ^17^ Institut de Génétique et de Biologie Moléculaire et Cellulaire (IGBMC), Illkirch-Graffenstaden, France; ^18^ Laboratoire de Diagnostic Génétique CHRU de Strasbourg, Strasbourg, France

## Abstract

Congenital nemaline myopathies are rare genetic disorders that typically manifest at birth or in childhood, with muscle weakness and respiratory distress. They are characterized by the presence of rod‐like structures on the muscle biopsy, or a mix of rods with cores, focal areas with disorganization of oxidative activity. Pathogenic variants in the *NEB* gene represent a main cause of these conditions. However, significant phenotypic variability exists, hampering disease prognosis, healthcare, and genetic counseling. This study is aimed at further characterizing the clinical and genetic features of nebulin‐related congenital myopathies and exploring genotype–phenotype correlations in a novel large cohort of patients. In a total of 49 patients from 48 families with nebulin‐related myopathies, a substantial proportion (23/49) exhibited a typical form of the disease, with childhood onset and preserved ambulation beyond 11 years of age. Respiratory insufficiency was observed in 33/49 patients, with a median age of 16 years for the initiation of ventilatory support. Facial muscle weakness was common (32/49), whereas severe bulbar symptoms were less frequent, with only 14/49 requiring nutritional support, all in cases where respiratory assistance was necessary. Patients with rods (*n* = 28) or core‐rods (*n* = 11) exhibited no significant clinical differences. Genetic analysis identified 76 *NEB* variants, including 7 recurrent, with a mutational hotspot detected between Exons 169 and 175 in 25/49 patients. Furthermore, our analyses suggest an association between the type of variant and five groups of patients with different levels of clinical severity. Additionally, our data raise the possibility that ophthalmoplegia may be preferentially observed in patients harboring variants located in Exon 143. Overall, these findings will contribute to facilitating and expediting genetic diagnosis in future cases with suspected *NEB* involvement, ultimately improving patient care and management.

## 1. Introduction

Congenital myopathies (CMs) are rare genetic disorders that significantly affect patient autonomy and often survival. They are typically characterized by congenital hypotonia, muscle weakness, delayed motor milestones, and normal to slightly elevated creatine kinase (CK) levels. CMs exhibit significant clinical heterogeneity, ranging from mild late‐onset forms to severe and life‐threatening early disease courses, exacerbated by additional features such as cardiomyopathy, respiratory failure, or skeletal deformities. Muscle biopsies from CM patients typically reveal structural abnormalities defining distinct CM subtypes [1, 2]. Nemaline myopathy (NM) is one of the major CM subtypes with an estimated incidence of 1 in 50 000 live births [3]. It is histologically characterized by fuchsinophilic protein inclusions forming rod‐like structures on muscle sections stained with Gomori′s trichrome [4]. To date, 14 genes have been identified associated with NMs [5], with recessive *NEB* variants constituting the predominant cause of the disorder [6, 7].

The *NEB* gene is almost exclusively expressed in skeletal muscle cells and encodes nebulin, a giant skeletal muscle protein that spans the actin thin filament in sarcomeres and is essential for filament integrity. Nebulin also plays a role in the Z‐disk organization and in the regulation of muscle contraction [8, 9]. It is composed of 22–29 tandem super‐repeats (SR), each consisting of seven simple repeats of 30– 35 amino acid residues. These simple repeats contain a conserved SDxxYK sequence motif, which is believed to mediate actin binding [10].

Despite advancements in DNA analysis methods and the routine use of next generation sequencing technologies with sophisticated variant filtering pipelines, the establishment of a genetic diagnosis for *NEB*‐related CM remains challenging. First, nonpathogenic variants accumulate in genes with increasing size, and *NEB* is composed of 183 exons and is among the largest genes of the human genome. Accordingly, the Genome Aggregation Database (gnomAD) lists more than 28,000 *NEB* variants including numerous missense changes in supposedly healthy individuals (https://gnomad.broadinstitute.org/gene/ENSG00000183091?dataset=gnomad_r4). Second, the presence of a repeat region containing a triplicated and almost identical set of 8 exons between Exons 82 and 105 challenges both sequencing and sequence analysis, particularly in terms of copy number variants [11]. Third, *NEB* transcripts undergo extensive alternative splicing to generate multiple *NEB* isoforms at different developmental stages, which further defies the prediction of the potential effect of *NEB* variants on nebulin function [11]. In fact, 42 of the 183 exons undergo alternative splicing: Exons 63–66, which are spliced together; Exons 82–105 within the triplicated region; Exons 143 and 144, which are mutually exclusive; and Exons 166–177, which undergo extensive alternative splicing, giving rise to a multitude of isoforms. Finally, recent reports of dominant inheritance patterns have challenged the classical view of *NEB*‐related myopathy that was formerly considered a recessive condition [12].

In addition to the genetic complexity, *NEB*‐related CM is also associated with major clinical and histological heterogeneity [13]. Indeed, the clinical presentations of affected individuals can range from severe congenital hypotonia, arthrogryposis, and respiratory failure increasing the risk of premature death to milder adult‐onset forms compatible with autonomous walking and normal life‐span [14]. Histologically, muscle biopsies from *NEB* patients may display cores in addition to the eponymous nemaline rods, and the concomitant occurrence of both histopathological features is referred to as a core‐rod myopathy [15].

Overall, the genetic, clinical, and histological heterogeneity characterizing *NEB*‐related CM significantly challenges genetic counselling and compromises the possibility to provide an accurate prognosis on disease development. To expand the knowledge on *NEB*‐related CM and contribute to a better understanding of the spectrum of clinical variability, muscle histopathology, and variant type and position, we thoroughly examined a cohort of 49 patients with a focus on interventional support and the establishment of a genotype–phenotype correlation.

## 2. Material and Methods

### 2.1. Patient Recruitment and Evaluation

Forty‐nine patients with clinical manifestations of NM and harboring causative variants in *NEB* were included in this multicentric study. Patients originated from Argentina, Benin, Chile, France, Paraguay, Sri Lanka, Syria, and Turkey, and all presented with either neonatal hypotonia or early‐ or late‐onset muscle weakness. Clinical data were retrospectively gathered from the patients′ medical records. The majority of patients (40/49) underwent muscle biopsies with subsequent histological analysis, and most were viewed by the Morphology Unit of the Myology Institute (Paris, France). Patients were categorized into seven homogeneous groups based on clinical severity as defined in a previously published classification [14] (Table 1).

**Table 1:** Clinical classification of *NEB*‐related CM (Modified from Lehtokari VL, et al. 2014).

Form of congenital myopathy	Clinical criteria
Severe	Onset at or before birth: absence of spontaneous movements, absence of autonomous respiration, or presentation with severe contractures or fractures at birth
Intermediate	Infantile onset characterized by an early requirement for ventilatory support and the inability to achieve independent sitting or walking, or wheelchair use before the age of 11 years
Typical	Onset in infancy with delayed but achieved motor milestones; slowly progressive or nonprogressive course. If required, wheelchair use after the age of 11 years
Childhood onset	Childhood onset
Adult onset	Adult onset
Distal myopathy	Mainly distal involvement at presentation
Atypical form	Unusual associated features (e.g., ophthalmoplegia, cardiomyopathy)

### 2.2. Ethical Statement

All patients or legal guardians provided written informed consents according to the declaration of Helsinki and its later amendments. The present study was approved by the IRB Comité de Protection des Personnes Est IV (DC‐2012‐1693).

### 2.3. DNA Sequencing and Data Analysis

Thirteen patients (P1–P10 and P36–P38) underwent exome sequencing as part of the French MYOCAPTURE next‐generation sequencing project aiming to uncover the genetic causes in families with undiagnosed myopathies [16]. DNA samples were processed using the SureSelect Human all Exon 50 Mb V5 capture library (Agilent, Santa Clara, United States), and enriched DNA fragments were subsequently exome‐sequenced on an Illumina HiSeq2500 (Illumina, San Diego, United States). One patient underwent genome sequencing (P11) in the Laboratory of Molecular Genetics in Dijon, France. The remaining 35 patients underwent routine panel sequencing encompassing known neuromuscular disorder genes in accredited diagnostic laboratories (academic molecular genetics laboratories in Grenoble and Strasbourg, and the Invitae genetic testing laboratory). Details of the genetic tests are provided in Table S1.

Sequence data were aligned to the GRCh37/hg19 reference genome and variants were filtered based on various criteria, including inheritance pattern, coverage, and read depth, as well as epidemiological data such as frequency in the gnomAD, literature research, and structural data. Depending on the gene involved, variants were ranked according to the clinical and histological characteristics of the patients. The potential pathogenic impact of the prioritized variants was predicted using the Alamut software (Sophia Genetics), incorporating multiple bioinformatics prediction algorithms such as SIFT and PolyPhen for missense, and MaxEntScan, NNSplice and Splice Site Finder for splice variants. The most convincing variants were subsequently confirmed by Sanger sequencing, and cosegregation with the disease was verified on DNA samples from available relatives. For selected synonymous or splice variants, RNA analysis was performed on patient muscle tissue to assess their functional impact (see File S2 for details). *NEB* variants were annotated according to GenBank NM_001271208.1 and NP_001258137.1 with +1 corresponding to the A of the ATG translation initiation codon.

### 2.4. Haplotype Analyses

For one recurrent *NEB* variant in our cohort, we conducted haplotype analyses to conclude on potential founder effect. Four microsatellite markers flanking the *NEB* gene—namely, D2S2277, D2S356, D2S2275, and D2S2299—were examined through PCR with fluorescently labelled primers, and the resulting amplicons were analysed on an automated ABI3100 sequencer (Thermofisher) to determine individual microsatellite length.

### 2.5. Statistical Analysis

The Shapiro–Wilk test was used to assess the normality of data distribution. Normally distributed continuous data were presented as mean ± standard deviation, whereas nonnormally distributed continuous data were expressed as median with interquartile range (IQR). Categorical data were summarized as frequencies and percentages. Depending on sample size, chi‐squared or Fisher′s exact tests were used for comparisons between patients with and without ventilatory support, as well as between patients with classical NM and those with core‐rod myopathy. Kaplan–Meier analysis was performed to estimate the age at initiation of ventilatory support, accounting for right‐censoring due to patients who had not yet required ventilatory support at last follow‐up. Patients with missing or incomplete data on ventilatory status were excluded from this analysis. Data analysis was performed using Prism 10 (GraphPad Software, San Diego, United States). A *p* value < 0.05 was considered statistically significant.

## 3. Results

### 3.1. General Clinical Characteristics

A total of 49 index cases from 48 families with CM caused by *NEB* variants enrolled in the present study. All patients were unrelated except for a mother and her daughter (P48 and P49) who shared distinct *NEB* variants. An overview of the clinical and histological characteristics of each patient is provided in Table S2.

**Figure 1:**
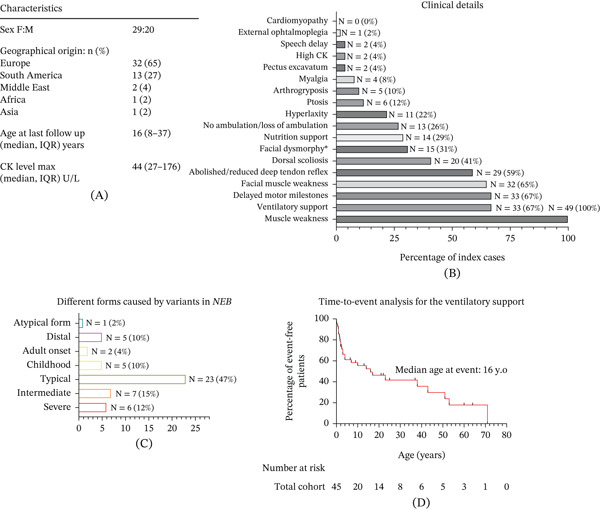
Detailed clinical characteristics of the cohort of patients with *NEB*‐related CM. (A) General characteristic. Abbreviations: CK, creatine kinase; F, female; IQR, interquartile range; M, male. (B) Frequency of clinical signs and symptoms. (C) Frequency of *NEB*‐related CM forms. (D) Time‐to‐event analysis for the ventilatory support. Abbreviation: y.o, years old.

Overall, we determined a male‐to‐female ratio of 1:1.5 (Figure 1A). A total of 51% of all patients were sporadic cases or singletons (25/49), whereas a family history with more than one affected member was noted in 10 families. Other families lacked available information. Parental consanguinity was reported in six families. With the exception of P34, a foetus with akinesia, arthrogryposis with clubfoot and clubhand, generalized oedema, pericardial, pleural, and ascitic effusions and intrauterine death, the median age at last clinical follow‐up was 16 years old (IQR: 8–37).

Across the cohort, several common clinical characteristics emerged (Figure 1B; Table S2). Muscle weakness was a consistent feature (*n* = 49), typically affecting both proximal and distal muscles, with rare cases manifesting a predominant involvement of distal muscles (P12, P18, P30, P31, and P41). CK levels were generally normal, with a median of 44 U/L (IQR: 27–176 U/L, reference range: 50–200 U/L), and mildly elevated in P17 (473 U/L) and P25 (600 U/L). Thirty‐eight patients (78%) achieved independent walking; however, only 29% of these patients reached this motor milestone within the first 17.5 months, considered as the normal developmental time window by the World Health Organization (WHO). Ten patients exhibited a limited walking perimeter and required assistance or a wheelchair for longer distances, and seven patients experienced a total loss of ambulation. Respiratory involvement resulting in restrictive insufficiency was frequent, necessitating invasive or noninvasive ventilation in 33 patients (67%). Most patients displayed myopathic facies characterized by hypomimia or facial hypotonia (*n* = 32). Conversely, only six patients presented with ptosis, either isolated (*n* = 5) or in combination with external ophthalmoplegia (*n* = 1). Skeletal deformities were also prevalent, with 20 patients (41%) presenting with scoliosis, including eight patients requiring correction surgery. Bulbar involvement was evident in 14 patients, leading to swallowing difficulties; fourteen patients (29%) required enteral nutritional support at a median age of 1 year (IQR: 0–9). Eleven patients (22%) exhibited hyperlaxity. Speech delay was noted for two patients (P9 and P11). No cardiomyopathy was reported.

### 3.2. Clinical Heterogeneity

Although the patients in our cohort displayed several common clinical features, we noted heterogeneity in terms of disease severity. Nearly half of the patients (23/49; 47%) presented with a typical phenotype, with onset of symptoms within the first year of life (Figure 1C). Among these 23 patients with the typical phenotype, none had congenital contractures, except for P27, who presented with bilateral clubfeet but remained ambulant and active at 17 years of age. All patients in this group achieved independent walking with a median age of 19 months (IQR: 18–19) and only three experienced loss of ambulation. Fourteen patients (61%) required ventilatory support, initiated at a median age of 4 years old (IQR: 2–16), and three patients (13%) needed nutritional support.

About one‐fourth of the cohort (13/49; 27%) presented with an intermediate or severe form of the disease. Six patients (12%) exhibited the severe form and manifested congenital hypotonia. Four of these patients had arthrogryposis, and four required ventilation at birth or died within the first 2 months of life. Seven patients (14%) had an intermediate phenotype with no ambulation or wheelchair use before the age of 11 years. All were ventilated before the age of three.

The remaining patients in our cohort exhibited either distal (*n* = 5, 10%) or milder forms, with the occurrence of first disease signs during childhood (*n* = 6) or adulthood (*n* = 2). Among these patients with childhood‐onset, P17 was classified as an atypical form owing to the presence of external ophthalmoplegia.

### 3.3. Ventilatory Support

A comparative overview of ventilated and nonventilated patients is provided in Table 2. Thirty‐three patients (67%) required invasive or noninvasive ventilation with initiation at a median age of 16 years (Figure 1D). Compared with nonventilated patients from our cohort, patients requiring ventilation showed more severe outcomes with significantly higher rates of nutritional support (*p* = 0.002). Although the proportion of patients requiring a wheelchair or presenting with scoliosis was higher among ventilated than nonventilated patients, these differences did not reach statistical significance (*p* = 0.07 and *p* = 0.18, respectively).

**Table 2:** Comparison of patients with and without ventilatory support.

	Ventilatory support (*n* = 33)	No ventilatory support (*n* = 15)	Fisher exact test (*p*)	Odds ratio (95% CI)
Sex F:M	19:14	9:6		
Wheelchair^a^	10/27	1/13	0.07	6.78 (0.77; 330.80)
Nutrition support	14/33	0/15	0.002	
Scoliosis^a^	16/27	4/13	0.18	3.17 (0.67; 17.89)
Age at last follow up (median, IQR); years	15 (8–45)	21 (9–25)		

Abbreviations: CI, confidence interval; IQR, interquartile range.

^a^ Patients older than 3 years.

### 3.4. Muscle Histology

**Figure 2:**
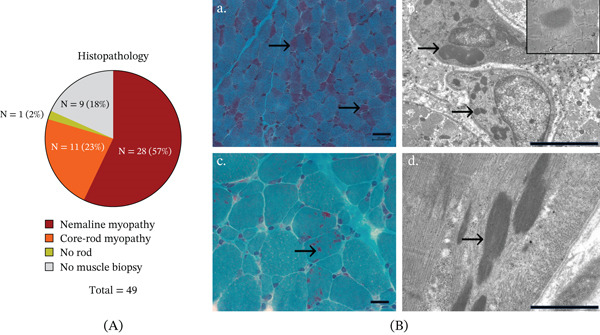
Detailed histological characteristics of the cohort of patients with *NEB*‐related CM. (A) Frequency of histopathological subtypes. (B) Representative muscle biopsy findings in nemaline myopathy (P1 and P8). (a and b) Muscle biopsy from a 1‐month‐old newborn with *NEB*‐related CM (P1). Muscle figures (a: GT, b: EM) showed marked variation in fiber size and clusters of nemaline bodies in the small muscle fibers (scale bar 20 *μ*m). EM analysis particularly revealed numerous rounded rod bodies, and rare cytoplasmic bodies as shown in the inset (Scale bar 3 *μ*m). (c and d) Muscle biopsy from a 1‐year‐old infant with *NEB*‐related CM (P8). Muscle figures (c: GT, d: EM) showed manifest variation in fiber size and scattered nemaline bodies in some muscle fibers (scale bar 20 *μ*m). EM analysis particularly revealed elongated rod bodies in several muscle fibers (scale bar 1 *μ*m). Abbreviations: EM, electron microscopy; GT, Gomori′s trichrome.

A muscle biopsy was conducted for the majority of patients (*n* = 40/49), with a median age at biopsy of 6 years old (IQR: 1–28). Morphological findings of the available muscle biopsies are detailed in Figure 2A. Histological examinations of the muscle biopsies frequently revealed features consistent with NM, characterized by the presence of subsarcolemmal or perinuclear rods as the predominant feature (*n* = 28/40; 70%). These rods typically appeared as rounded, ovoid bright red elements on modified Gomori′s trichrome staining. Representative images from two patients (P1 and P8) are provided in Figure 2B. Additional electron microscopy was performed on 16/28 patients and consistently confirmed the presence of electron‐dense structures corresponding to rods. ATPase staining demonstrated a predominance of Type I fibers in 15 patients (54%), with an exclusive presence of Type I fibers in three patients (P19, P44, and P45).

For 11 cases (28%), rods were accompanied by cores, visualized either on oxidative staining or on electron microscopy, defining core‐rod myopathy. No statistical difference between classical NM and core‐rod myopathy was observed regarding wheelchair use (*p* = 1), nutritional support (*p* = 0.45), respiratory support (*p* = 0.45), and scoliosis (*p* = 0.55).

For a single patient (P40), histological examination of the muscle biopsy did not reveal NM‐typical rods or cores but disclosed rather nonspecific signs of CM including predominance of Type I fibers and fiber size variability.

### 3.5. Molecular Diagnosis

**Figure 3:**
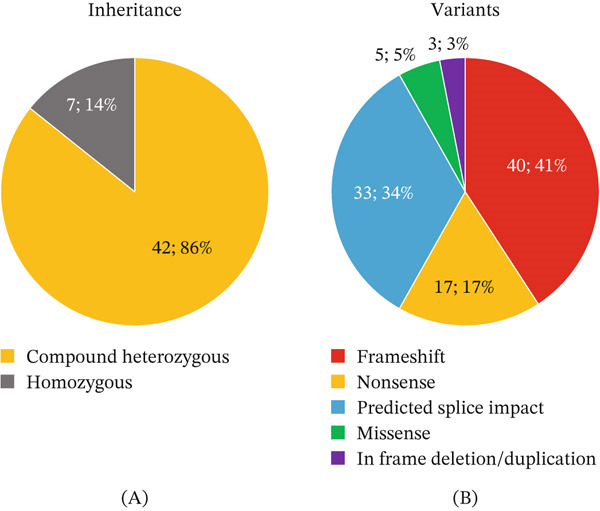
Genetic overview of *NEB*‐related CM patients. (A) Distribution of inheritance patterns. (B) Distribution of variant types.

Exome and genome sequencing were, respectively, performed for P1–P10 and for P11, whereas targeted gene sequencing was employed for P12–P49, revealing the presence of autosomal recessive *NEB* variants in all individuals (Table S1). Among the patients, 42 harbored two distinct variants, with segregation analysis available in 29 families confirming a trans configuration; seven patients exhibited homozygosity (Figure 3A). Consanguinity was reported in six families, three of which harbored homozygous variants, and three carried two distinct heterozygous variants. Segregation analysis was possible in two of these latter families and confirmed the trans configuration. Causative variants were consistently confirmed by Sanger sequencing, and compound heterozygosity was validated through segregation analysis on available parental DNA samples (*n* = 29/42 families). A total of 98 causative variants were identified, and their distribution, including recurrent variants, is depicted in Figure 3B. The majority (58%) was predicted to result in premature stop codon and potentially nonsense‐mediated mRNA decay (NMD), including nonsense variants (17%) and small out‐of‐frame insertions or deletions predicted to induce frameshifts (41%). Among the remaining variants, 33 (34%) were predicted to affect splicing, comprising 29 splice site and 4 synonymous variants predicted to alter normal splicing either by impairing the consensus splice site or by activating a cryptic splice site. RNA analysis was performed on transcripts originating from muscle biopsy samples of P18, P44, and P45 to evaluate the impact of the identified variants on splicing (Figure S1). Additionally, five missenses (5%) and three in‐frame deletion/duplications (3%) were documented.

In total, 76 distinct causative variants were identified, of which 42 (55%) were previously unreported. The majority of these variants were unique, with only seven (9%) being recurrent. Of these, five were shared by a maximum of two families each, whereas the c.24218C>A (p.Ser8073∗) was shared by three families, and the c.24294_24297dup (p.Glu8100Serfs∗5) by eight families. All but one of the families harboring the c.24294_24297dup variant were from Argentina or Paraguay, but none declared a known relationship with the other affected families. The c.24218C>A (p.Ser8073∗) was detected in three patients (P14, P16, and P31) at the heterozygous state either in combination with a splice site (P14) or another nonsense (P16 and P31). All three presented with childhood disease onset, preserved ambulation until adulthood, and a requirement for noninvasive ventilation. In contrast, the c.24294_24297dup (p.Glu8100Serfs∗5) was associated with greater phenotypic heterogeneity, with three patients showing intermediate forms (P7, P8, and P40), four presenting with typical forms (P6, P10, P38, and P39), and one displaying a distal form of the disease (P41). This variant was previously reported in patients with NM [14, 17, 18]. Analysis of microsatellite repeats performed in Families 6, 8, and 10 revealed no common haplotype, excluding a close relationship between the families and suggesting that the c.24294_24297dup variant may have occurred independently or reflect an ancient founder effect. (Figure S2).

**Figure 4:**
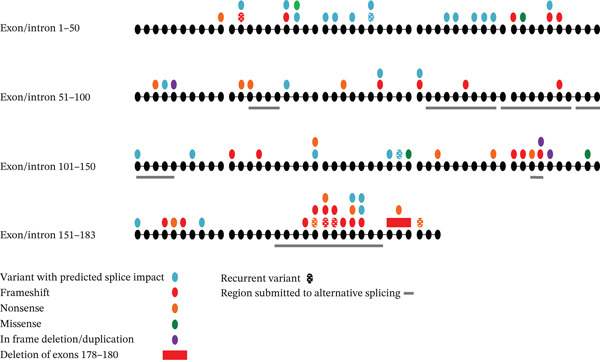
Genomic distribution of the analysed *NEB* variants. *NEB* exons (1–183, NM_001271208.1) and introns are represented as spheres connected by lines, respectively. Variants identified in each patient are displayed above the corresponding exons, with each sphere representing a single variant. Shaded spheres indicate recurrent variants.

The identified *NEB* variants in our cohort are distributed throughout the gene (Figure 4), with a predominant localization in the Exons 169–175, i.e. in an alternatively spliced region spanning Exons 166–177. Indeed, 25 patients (51%) harbored a causative variant within this region, including 18 frameshifts, 3 nonsense, and 3 splice‐site variants. This finding reveals a previously unrecognized mutational hotspot.

### 3.6. Exploratory Genotype–Phenotype Relationships in *NEB* Patients

Despite the clinical heterogeneity observed in this cohort of *NEB* patients, some recurrent patterns suggest genotype–phenotype correlations.

**Figure 5:**
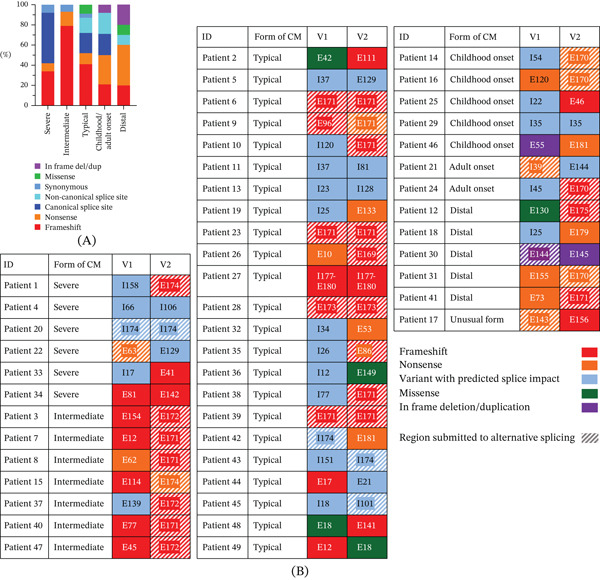
Genetic characteristics of patients with *NEB*‐related CM. (A) Distribution of variant types compared to the clinical severity. P17, who presented with an unusual phenotype (ophthalmoplegia), was included in the childhood/adult‐onset group for this analysis, as symptom onset occurred during childhood. (B) Distribution of the analysed *NEB* variants based on whether the exons undergo alternative splicing. Abbreviations: CM, congenital myopathy; E, exon; I, intron; V, variant.

First, the type and distribution pattern of the identified variants appear to be associated with disease severity (Figure 5A). Variants without a predicted impact on protein expression such as missense and in‐frame deletions/duplications were exclusively found in patients with mild or moderate disease signs including typical (P2, P36, P48, and P49) or distal forms (P12 and P30), or in a patient with childhood onset (P46). None of these variants was detected in patients with severe or intermediate disease forms. Conversely, all patients with an intermediate phenotype (*n* = 7) exhibited a combination of two frameshift and/or nonsense variants, which are expected to strongly impair protein expression. One of these patients (P37) carries compound heterozygous frameshift (c.24373del; p.Arg8125Glufs∗55) and synonymous (c.20928G>T; p.Gly6976=) variants. However, RT‐PCR and Sanger sequencing on muscle mRNA disclosed a major impact of the synonymous variant on splicing, resulting in an out‐of‐frame partial exclusion of Exon 139 and the occurrence of a premature stop codon (p.Gly6976Aspfs∗19). The pathogenic impact of the splice site variants appears to depend on their precise position. Nucleotide substitutions affecting canonical acceptor or donor splice sites were more frequently detected in patients with severe disease forms (P1, P4, P20, and P33), whereas noncanonical splice site variants were restricted to milder phenotypic forms potentially as a result of a partial effect on splicing.

Second, *NEB* variants in ubiquitously expressed exons or in exons subject to alternative splicing may contribute differently to phenotypic severity (Figure 5B). Nearly all patients carrying a combination of two truncating variants (frameshift and/or nonsense) carry at least one variant in an alternative exon (*n* = 16/18). This suggests that high‐impact variants are less pathogenic in alternative exons and that the occurrence of two *NEB* variants in ubiquitously expressed exons is not tolerated and incompatible with life. Accordingly, the gnomAD does not list truncating variants in *NEB* at the homozygous state in the healthy population (https://gnomad.broadinstitute.org, accessed December 20, 2025). This is also supported by the highly severe phenotype of P34 harboring the compound heterozygous truncating variants c.12160del (p.Trp4054Glyfs∗12) and c.21278_21280delinsA (p.Arg7093Lysfs∗11) in ubiquitous exons. Indeed, this fetus exhibited fetal akinesia, arthrogryposis, and hydrops fetalis, and the pregnancy was terminated at 17 weeks of gestation. Of note, a twin from a previous pregnancy in the same family had also died from unexplained hydrops fetalis. Although no genetic testing was performed, it is likely that the undiagnosed patient carried the same high‐impact variants.

## 4. Discussion

*NEB*‐related myopathies constitute a group of heterogeneous conditions with a large spectrum of manifestations and severity. The physiopathological mechanism is primarily related to a nebulin deficiency, leading to sarcomere disorganization, changes in thin filaments length, and subsequent reduction in force generation [19–21]. The primary goal of this study was to provide an extended characterization of the clinical, histological, and genetic features of patients from 48 families with *NEB*‐related NM in order to optimize patient management and improve genetic diagnosis and counselling. Forty‐three patients were not previously reported in the literature. Several key clinical characteristics emerged. In addition to consistent muscle weakness, we observed a high prevalence of respiratory insufficiency, with 67% of patients requiring assisted ventilation and facial involvement in 65% of the patients. Although respiratory impairment was frequent, its onset varied considerably, with a median age for ventilatory support initiation at 16 years. When present, respiratory impairment was often associated with a higher rate of comorbidities, such as the need for nutritional support. Patients with *NEB*‐related CM also differed in their muscle histopathology, with 70% exhibiting nemaline rods and 28% showing both rods and cores on their biopsy. Overall, 37% of the identified *NEB* variants had not been previously reported. Furthermore, we were able to delineate a genotype–phenotype correlation depending on both variant type and localization, although additional underlying mechanisms remain to be identified.

### 4.1. Clinical Signs in *NEB*‐Related Myopathy: Shared Features and Differences

All patients included in this cohort presented with *NEB*‐related CM, but we noted a marked phenotypic heterogeneity across the families. A large number of patients (47%) presented with a typical disease picture encompassing infantile onset, delayed but achieved motor milestones and ambulation preserved beyond 11 years of age. In contrast, 27% presented with a more severe phenotype including arthrogryposis, neonatal respiratory distress requiring ventilatory support, absence of independent sitting or walking, or early use of a wheelchair. The same proportion of patients (26%) exhibited milder forms with childhood or adulthood disease onset, sometimes restricted to distal muscle involvement. The prevalence of the typical disease form is consistent with findings from several other studies [14, 22, 23]. A potential limitation of our study is the nonrepresentative nature of the cohort, with 92% of patients originating only from two distinct geographic regions: Europe (mainly France) and South America. However, the comparable ratio of typical, intermediate, and severe disease forms in our study and previous reports indicates that the onset, course, and severity of *NEB*‐related CM are independent of the geographic origin and population [14, 22, 23].

Across the different clinical forms of the disease, it is important to note that patients with *NEB*‐related CM share common clinical features. First, the most frequent comorbidity was restrictive respiratory insufficiency requiring ventilatory support, observed in 67% of patients. However, initiation of assisted ventilation was highly variable in age, ranging from birth to 71 years. In contrast, severe bulbar involvement (29% requiring nutritional support) and marked muscle weakness leading to wheelchair dependence (26% who lost ambulation or never acquired independent walking) were less prevalent. These findings are in accordance with a previous report describing ventilatory support in 55% of patients with *NEB*‐related NM, compared with 32% requiring G‐tube placement [24]. Due to the retrospective nature of our study, no standardized swallowing assessment scale was used. Only the presence or absence of enteral support was considered, which does not rule out the possibility of undocumented minor bulbar symptoms. The comparison between patients with and without ventilatory support evidenced that the need for ventilation is concomitant with a higher frequency of nutritional support. These findings are in agreement with a previous report [24], and are helpful to adapt patient monitoring with particular attention to bulbar function when ventilation is required.

Second, facial muscle involvement ranging from localized weakness (e.g., open mouth or orbicularis oculi weakness) to more generalized involvement with complete facial diplegia was identified in 65% of our patients, representing another common trait in the cohort. This was more frequent than orofacial deformities, such as elongated face or high‐arched palate, which were reported in only 31% of the patients. Of note, ptosis, a common feature of several CMs, was only observed in 12% of our patients. Strikingly, hyperlaxity was present in 22% of the patients from the cohort, highlighting that it should be considered as a sign for *NEB*‐related CM in particular, especially in combination with muscle weakness. This was already described for other CMs such as multiminicore or central core diseases [25]. When not properly evaluated, hyperlaxity can lead to diagnostic delays and errors as illustrated for P31, who was initially suspected of having Ehlers–Danlos syndrome. Except for P9 and P11, no speech delay was observed in our cohort. In P9, this was most likely attributable to central hypothyroidism, an unexpected confounding factor, with improved following thyroid hormone replacement. In P11, speech delay was more likely related to orofacial muscle involvement associated with persistent articulation difficulties.

Recognizing these clinical characteristics is crucial as they can guide clinicians and geneticists towards an often challenging diagnosis and can help tailor patient monitoring, with a particular focus on respiration. MRI data were not available for our patients and would certainly have provided further support in the diagnostic process. Indeed, a characteristic pattern of muscle and muscle group involvement has been documented in NM and was successfully used to differentiate between *NEB*‐, *ACTA1*‐, and *TPM3*‐related NM [26].

### 4.2. Muscle Histology in *NEB*‐Related Myopathy: Shared Features and Differences

On 40 available muscle biopsies, all patients except P40 exhibited rods when available. Rods are a nearly consistent histological marker of *NEB*‐related CM [14]. With the exception of P4 and P40, rods were persistently detected using Gomori′s trichrome staining and were also visible by electron microscopy on biopsies undergoing ultrastructural investigations. In contrast, rods were undetectable by histological stains on muscle samples from the neonatal P4 despite a severe phenotype, and only electron microscopy revealed the presence of rods in small numbers. In P40 presenting with an intermediate clinical form and nonspecific signs of CM (predominance of Type I fibers and fiber size variability), immunohistochemical staining of muscle sections failed to disclose rods whereas electron microscopy was not performed. Both examples underscore the critical and complementary role of ultrastructural analyses in the diagnostic process of NM, especially if routine histology approaches do not reveal distinctive anomalies on muscle biopsies [13].

For another patient (P24) with an adult‐onset form, repeated muscle biopsies at ages 35, 37, and 40 revealed rods on Gomori′s trichrome staining and electron microscopy solely in the most recent biopsy. The biopsies were taken from different sites (soleus at age 35 and deltoid at ages 37 and 40). This case highlights the challenge of detecting rods in milder clinical forms. This was reported for distal forms, where rods may be difficult to detect with routine light microscopy and may be inconspicuous or even absent on electron microscopy [27].

In 70% of patients who underwent muscle biopsy, rods were observed as the only histopathological sign, whereas rods in combination with cores were observed in 28% of the cases. This shows that the routine use of oxidative staining to uncover cores in muscle sections from NM patients is crucial for an accurate classification. Although the mechanism of core formation in *NEB*‐related myopathy is not fully elucidated, it has been observed that many patients harboring the Exon 55 deletion showed cores on muscle biopsy [14, 28], as observed for P46.

In addition to rods and/or cores, Type I fiber predominance was observed in the majority of patients (90%) in whom ATPase staining was reported, with Type I uniformity noted in three patients.

### 4.3. *NEB* Sequencing Reveals New and Relevant Insights for Molecular Diagnosis

In the 49 patients, exome, genome, or targeted gene sequencing identified causative *NEB* variants either homozygous or heterozygous. Seventy‐six distinct *NEB* variants were identified, including 42 previously unreported (55%). All variants were classified according to ACMG standards and guidelines [29]. With the exception of seven patients harboring variants of unknown significance (VUS), all individuals carried pathogenic or likely pathogenic variants. Six patients were previously reported in the scientific literature [13, 14, 16, 30], and were included in the present work with a more detailed clinical, histological, and genetic characterization.

In accordance with prior reports, truncating *NEB* variants including frameshift and nonsense are more common (58%) in our cohort than variants affecting either canonical splice sites or adjacent splice relevant regions (30%) [14, 22]. We also identified the most common c.7431+1919_7536+374del (p.Arg2478_Asp2512del) variant resulting in the exclusion of *NEB* Exon 55 in the mRNA [31], whereas large dominant *NEB* deletions were not detected. This is consistent with other studies describing a minor contribution of deletions to *NEB*‐related CMs [12, 32], but may also reflect shortcomings of routine diagnostic techniques to efficiently discern larger structural aberrations within the *NEB* gene.

For five patients, nonconsensus splice site variants were considered relevant due to elevated splicing prediction scores by in‐silico models. However, muscle samples allowing RNA study to validate the impact of these variants were not available, so they were considered as VUS. In two patients, a missense VUS was identified and found to segregate with disease in both the symptomatic mother (P48) and her daughter (P49). Moreover, each affected individual also carried a distinct frameshift variant in trans with the shared VUS, consistent with pseudodominant inheritance. For all these patients, none of the VUS have been reported at the homozygous state in healthy individuals in the gnomAD [33], and all occurred in combination with either a pathogenic (*n* = 6) or a likely pathogenic *NEB* variant (*n* = 1). All these patients had a clinical presentation compatible with CM, and all displayed rods or rods and cores on muscle sections, reinforcing the implication of these VUS in the disease. Finally, all patients underwent exome or gene panel sequencing, which excluded common differential diagnoses such as *ACTA1*‐ or *TPM3*‐related NM [34, 35] and *RYR1*‐related core‐rod CM [36]. Taken together, these observations support a potential contribution of these VUS to the disease.

Regarding variant distribution, we observed an apparently even distribution throughout the gene, with variants identified in 59 of the 183 exons constituting the *NEB* gene. However, 25 patients (51%) harbored at least one causative variant in a region spanning Exons 169–175 which is submitted to alternative splicing. A similar enrichment in this region has been observed in previously reported large cohorts [14, 24]. Importantly, Exons 169–175 fall within the C‐terminal portion of nebulin. This region has been implicated in Z‐disk anchorage and interactions with key structural proteins, including desmin [37–39] and CapZ [40], supporting its functional importance [8]. Taken together, these findings support this region as a mutational hotspot.

Noteworthy, the c.24294_24297dup variant, corresponding to p.Glu8100Serfs*5, within this region was identified in eight unrelated families and has been previously reported in NM patients [14,17,18]. Seven of these families were diagnosed in Argentina and one in France, indicating a possible founder effect, but microsatellite repeat analysis conducted on three families did not reveal a shared haplotype.

### 4.4. Ophthalmoplegia: A Rare and Scarcely Reported Feature in *NEB*‐Related Myopathy

In our entire cohort, only a single adult patient presented with ophthalmoplegia (P17), an uncommon finding in *NEB*‐related CM. This patient was the only one carrying a heterozygous nonsense variant in the alternatively spliced Exon 143. To our knowledge, only one similar observation has been reported in the literature, also involving a heterozygous nonsense variant in Exon 143 [14]. These observations may suggest a possible association between Exon 143 variants and ophthalmoplegia that remains to be confirmed by the characterization of additional patients.

Exons 143 and 144 are mutually exclusive and never occur conjointly in *NEB* transcripts. Isoforms containing either Exon 143 or Exon 144 are known to have distinct expression profiles during development and in adult skeletal muscles, and consequently, variants in one or both exons result in a differential pattern of muscle weakness [11, 41, 42]. With respect to the rare ophthalmoplegia phenotype in *NEB* patients, it is plausible to assume that isoforms containing Exon 143 have a higher expression in oculomotor muscles either during and/or after development to ensure functional maintenance.

### 4.5. Genotype–Phenotype Correlation: New Clues and Limitations

In this study, we explored potential associations between variant characteristics and the phenotypic variability observed in *NEB* patients.

Concerning variant types, those with only minor predicted effects on protein expression were generally associated with milder phenotypes. In accordance with previous reports [14, 43, 44], missense variants were only observed in patients with distal muscle involvement or slowly progressive muscle weakness in our cohort, even in compound heterozygosity with truncating *NEB* variants. Similarly, noncanonical splice‐site variants, which are expected and predicted to partially interfere with normal splicing, were only associated with typical, late‐onset, and distal disease forms. In contrast, canonical splice‐site variants, predicted to strongly impair splicing and mRNA steady state levels, could be found in patients with more severe phenotypes, such as congenital forms involving hypotonia, arthrogryposis, and respiratory insufficiency [45]. Interestingly, all identified canonical splice‐site variants affected in‐frame exons, suggesting that the exclusion of single exons may have pathogenic consequences comparable to truncating variants. Functional studies would be required to assess the impact of these variants on mRNA processing, stability, and protein expression. However, establishing a direct correlation between predicted variant impact and actual nebulin protein levels remains challenging. Previous immunohistochemical studies using antibodies targeting different nebulin domains have reported variable and sometimes discordant results, likely reflecting both the complexity of *NEB* alternative splicing and the diversity of resulting isoforms [46–48].

For truncating variants encompassing nonsense and frameshift, phenotypic severity appeared to vary with the ubiquitous or alternative nature of the affected exons. All patients carrying a combination of two truncating variants, with at least one being located in a region subject to alternative splicing, exhibited mild to intermediate disease forms. Conversely, P34, who carried two truncating variants in constitutively expressed exons, presented with the most severe phenotype and manifested fetal akinesia, arthrogryposis, and hydrops fetalis. This is in alignment with previous case reports [14, 49] and suggests that two truncating *NEB* variants in ubiquitously expressed exons, likely leading to systematic mRNA degradation and strongly reduced protein expression, are incompatible with life. However, P27 somewhat represents an exception to this apparent trend. Despite the presence of the homozygous c.24870+22_25255+12del frameshift deletion encompassing the ubiquitously expressed Exons 178–180, P27 presented with a typical NM phenotype and still maintains ambulation at 17 years old. A similar observation was made by Lehtokari et al. [14], who reported a patient with homozygous frameshift in Exon 180 and a severe, yet viable, phenotype. This can possibly be explained by the comparably low effect of 3 ^′^ truncating variants on mRNA stability and degradation or the use of a cryptic splice site restoring the phase. Alternatively, these terminal exons might not be included in all *NEB* isoforms, contradicting current assumptions. However, these mechanisms remain to be experimentally validated.

Although our findings point toward potential genetic contributors to phenotypic variability, several limitations should be acknowledged. First, establishing genotype–phenotype correlations is challenging, as most *NEB* patients in general and in our cohort carry two different heterozygous variants. For instance, the c.25288C>T (p.Arg8430∗) nonsense variant paired with a noncanonical splice‐site variant was found in a patient (P42) with typical NM picture and rods on the muscle biopsy, whereas the same variant in combination with Exon 55 deletion was identified in a patient with late‐onset core‐rod myopathy. These observations illustrate the difficulty in attributing specific phenotypic features to individual variants. Second, significant interfamilial variability was observed in patients carrying the same *NEB* variants. By way of example, the unrelated P6 and P39 carry the homozygous c.2444_24447del (p.Pro8149Serfs∗30) nonsense variant. However, P39 exhibits a more severe phenotype requiring ventilatory support at 17 months compared with 10 years in P6. P39 also presented with scoliosis, which is absent in P6. This phenotypic variability suggests that modifier genes and/or environmental factors may influence disease severity.

## 5. Conclusions

This study contributes to a better understanding of *NEB*‐related CM and provides an integrated view on clinical, histopathological, and genetic data from one of the largest *NEB* cohorts encompassing 49 patients. In total, we identified 76 causative variants including 42 novel variants, thereby expanding the clinical and genetic spectrum of *NEB*‐related CM. Our findings also highlight potential genotype–phenotype relationships, although these remain to be further substantiated by functional investigations. We expect that the present work will facilitate genetic diagnosis of unresolved families clinically and histologically suggestive of *NEB* and will improve health management for already diagnosed patients.

## Funding

This study was supported by the IdEx Unistra, ANR‐10‐IDEX‐0002; the SFRI‐STRAT′US project, ANR‐20‐SFRI‐0012; the EUR IMCBio, ANR‐17‐EURE‐0023; the Horizon Europe program from European Union, COMPASS‐NMD 101080874; the AFM‐Téléthon, 23933; the Les Enfants Malades à l′Hôpital; the Inserm poste d′accueil fellowship; the Prix Coup de Pouce from the French Society of Myology; and the FONDECYT, #1110159.

## Conflicts of Interest

The authors declare no conflicts of interest.

## Supporting Information

Additional supporting information can be found online in the Supporting Information section.

## Supporting information


**Supporting Information 1** Table S1: Genetic characteristics for probands from 48 families with *NEB*‐related CM.

**Supporting Information 2** Table S2: Clinical and histological characteristics for probands from 48 families with *NEB*‐related CM.

**Supporting Information 3** File S1: Detailed methodology for transcript analysis used to assess the pathogenicity of splice site or synonymous variants.

**Supporting Information 4** Figure S1: Transcript analysis of three patients (P18, P44, and P45) to evaluate the pathogenicity of splice site or synonymous variants. Figure S2: Haplotypes of families 6, 8, and 10 for the duplication *NEB*: c.24294_24297dup (p.Glu8100Serfs∗5).

**Supporting Information 5** File S2: Collaborators from the NEB Study Group, listed in alphabetical order.

## Data Availability

The data that support the findings of this study are available from the corresponding author upon reasonable request.
