# Supplementary material for: Clinical, Histological, and Genetic Characterization of a Large Cohort of 49 Patients With Nebulin‐Related Congenital Myopathy
Source: Hum Mutat. 2026 Aug 17;2026:4385685. doi: 10.1155/humu/4385685 (PMC13478792; doi:10.1155/humu/4385685)
Supplement: Supplementary file 1 [file HUMU-2026-4385685-s003.docx]

**Supplementary Methods.**

**Transcript analysis**. mRNA was extracted from muscle biopsies *(NucleoSpin RNA Set for NucleoZOL, Macherey-Nagel)* and cDNAs were obtained by retro-transcription *(Transcriptase inverse Transcriptor, Sigma-Aldrich)*. cDNAs were amplified by PCR using 9 pairs of overlapping primers with KOD DNA Polymerase *(Sigma-Aldrich)* during 45 cycles. Amplicons were fragmented and sequenced on a S5 platform after library preparation *(NEBNext^®^ Library Quant kit for Illumina^®^, Biolabs)*. FASTQ of *NEB* transcripts were filtered and aligned using custom pipeline as previously described (Géraud J, et al. J Med Genet. 2020.). Reads and splice junctions were mapped using the RNASTAR software (Dobin A, et al. Bioinformatics. 2013.). Transcript sequences and splice junctions were visualized using IGV v.2.3.72. Transcript sequences were translated using Expasy translate tool.
