# Supplementary material for: Clinical, Histological, and Genetic Characterization of a Large Cohort of 49 Patients With Nebulin‐Related Congenital Myopathy
Source: Hum Mutat. 2026 Aug 17;2026:4385685. doi: 10.1155/humu/4385685 (PMC13478792; doi:10.1155/humu/4385685)
Supplement: Supplementary file 2 [file HUMU-2026-4385685-s004.docx]

**Supplementary Figure 1: Transcript analysis.**

Transcript analysis of three patients (P18, P44, and P45) to evaluate the pathogenicity of splice site or synonymous variants. **A:** Variant table summary. **B-D:** Sashimi plots showing aberrant splicing in patients P18, P44 and P45.

**Supplementary Figure 2: Haplotype analysis.**

Haplotypes of families 6, 8 and 10 for the duplication *NEB*:c.24294_24297dup (p.Glu8100Serfs*5).

Shaded cases: alleles associated with the pathogenic variant.
