# Supplementary material for: Clinical, Histological, and Genetic Characterization of a Large Cohort of 49 Patients With Nebulin‐Related Congenital Myopathy
Source: Hum Mutat. 2026 Aug 17;2026:4385685. doi: 10.1155/humu/4385685 (PMC13478792; doi:10.1155/humu/4385685)
Supplement: Supplementary file 3 [file HUMU-2026-4385685-s005.docx]

Collaborators from the NEB Study Group, listed in alphabetical order.

| **Collaborators** | **Affiliated institution** |
| --- | --- |
| Dr Christine Barnerias | Reference Center for Neuromuscular Disorders, Neuropediatric Department, Hôpital Necker Enfants Malades, Paris, France. |
| Dr Claude Cances | Reference Center for Neuromuscular Disorders, Pediatric Clinical Research Unit/Pediatric Multi-Thematic Module CIC 1436, Neuropediatric Department, Toulouse University Hospital, Toulouse, France. |
| Dr Jean-Baptiste Chanson | Reference Center for Neuromuscular Disorders, Neurology Department, Strasbourg University Hospital, Strasbourg, France. |
| Dr Anne Croue | Laboratoire d'anatomo-pathologie, CHU Angers, Angers, France. |
| Dr Jean-Marie Cuisset | Reference Center for Neuromuscular Disorders, Lille University Hospital, Lille, France |
| Dr Florence Demurger | Service de Génétique, Centre Hospitalier de Vannes, Vannes, France. |
| Pr Isabelle Desguerre | Reference Center for Neuromuscular Disorders, Neuropediatric Department, Hôpital Necker Enfants Malades, Paris, France. |
| Dr Klaus Dieterich | Service de Génétique, Centre Hospitalier de Grenoble, Grenoble, France. |
| Dr Nicolas Dondaine | Laboratoire de Diagnostic Génétique, CHRU de Strasbourg, Strasbourg, France. |
| Dr Julien Durigneux | Reference Center for Neuromuscular Disorders, Neuropediatric Department, Angers University Hospital, Angers, France. |
| Dr Theresinha Evangelista | Department of Neuropathology, Sorbonne University, APHP, Pitié-Salpêtrière Hospital, Paris, France. |
| Dr Antoinette Gelot | Service d'anatomie Pathologique, Hôpital Trousseau APHP, Paris, France. |
| Dr Arnaud Isapof | Reference Center for Neuromuscular Disorders, Neuropediatric Department, Hôpital Trousseau, Assistance Publique-Hôpitaux de Paris, Paris, France. |
| Dr Anne-Laure Kaminski | Reference Center for Neuromuscular Disorders, Department of Neurology, Angers University Hospital, Angers, France. |
| Dr Emeline Lagrange | Service de Neurologie, Centre Hospitalier Universitaire de Grenoble, Grenoble, France. |
| Dr Franck Letournel | Neurobiology and Neuropathology Unit, Department of Pathology, CHU d'Angers, Angers, France. |
| Dr Benoit Lhermitte | Service d'anatomie Pathologique, CHU Strasbourg, Strasbourg, France. |
| Dr Thierry Maisonobe | Department of Neuropathology, Sorbonne University, APHP, Pitié-Salpêtrière Hospital, Paris, France. |
| Dr Martial Mallaret | Service de Neurologie, Centre Hospitalier Universitaire de Grenoble, Grenoble, France. |
| Dr Véronique Manel | CMR Neuromusculaire, HFME, HCL, Bron, France. |
| Pr Sylvie Manouvrier-Hanu | Service de Génétique, Centre Hospitalier Universitaire de Lille, Lille, France. |
| Pr Marie-Laure Martin-Negrier | Department of Pathology, University Hospital of Bordeaux, Pellegrin Hospital, Place Amélie Raba Léon, Bordeaux, France. |
| Dr Sandra Mercier | Medical Genetics Department, Nantes University, CHU Nantes, F-44000 Nantes, France |
| Dr Marie Christine Nougues | Reference Center for Neuromuscular Disorders, Neuropediatric Department, Hôpital Trousseau, Assistance Publique-Hôpitaux de Paris, Paris, France. |
| Dr Caroline Perriard | Department of Paediatric, Nice University Hospital, Nice, France |
| Dr Anne Pervillé | Hôpital d'Enfants - ASFA, F-97404, Saint Denis, La Réunion, France. |
| Dr Céline Poirsier | Department of Genetics, Reims University Hospital, Reims, France. |
| Pr Sabrina Sacconi | Université Côte d’Azur, Peripheral Nervous System and Muscle Department, Centre Hospitalier Universitaire de Nice, Pasteur 2 Hospital, 30 voie Romaine CS 51069, Nice, France |
| Dr Marco Spinazzi | Neuromuscular Reference Center, Department of Neurology, CHU d'ANGERS/ INSERM U1083, Angers, France. |
| Dr Nathalie Streichenberger | INMG-PGNM, UMR CNRS 5261 - INSERM U1315, Université Lyon 1, Lyon, France; Département d'Anatomo-Pathologie, Groupement Hospitalier Est, Hospices Civils de Lyon, Lyon, France. |
| Pr Ana-Lia Taratuto | Neuropathology and Neuromuscular Diseases Laboratory, Buenos Aires, Argentina. |
| Dr Vincent Tiffreau | CHU Lille Neuromuscular Reference Center, F-59000, Lille, France, URePSSS, EA 7369, Univ Lille Nord de France, F-59000, Lille, France |
| Dr Clémence Vanlerberghe | Univ. Lille, CHU Lille, ULR 7364-RADEME - Maladies RAres du DÉveloppement embryonnaire et du Métabolisme, Lille, France. |
